# A systems-level approach reveals new gene regulatory modules in the developing ear

**DOI:** 10.1242/dev.148494

**Published:** 2017-04-15

**Authors:** Jingchen Chen, Monica Tambalo, Meyer Barembaum, Ramya Ranganathan, Marcos Simões-Costa, Marianne E. Bronner, Andrea Streit

**Affiliations:** 1 Department of Craniofacial Development and Stem Cell Biology, King's College London, London SE1 9RT, UK; 2 Division of Biology and Biological Engineering, California Institute of Technology, Pasadena, CA 91125, USA

**Keywords:** Auditory system, Cell fate, Chick, Embryo, Hearing, Placode, Transcription factor

## Abstract

The inner ear is a complex vertebrate sense organ, yet it arises from a simple epithelium, the otic placode. Specification towards otic fate requires diverse signals and transcriptional inputs that act sequentially and/or in parallel. Using the chick embryo, we uncover novel genes in the gene regulatory network underlying otic commitment and reveal dynamic changes in gene expression. Functional analysis of selected transcription factors reveals the genetic hierarchy underlying the transition from progenitor to committed precursor, integrating known and novel molecular players. Our results not only characterize the otic transcriptome in unprecedented detail, but also identify new gene interactions responsible for inner ear development and for the segregation of the otic lineage from epibranchial progenitors. By recapitulating the embryonic programme, the genes and genetic sub-circuits discovered here might be useful for reprogramming naïve cells towards otic identity to restore hearing loss.

## Abstract (precis)

**Summary:** Transcriptome analysis and knock down of select transcription factors reveals a genetic hierarchy as cells become committed to inner ear fate.

## INTRODUCTION

In vertebrates, the entire inner ear arises from the otic placode, a simple epithelium next to the hindbrain, which invaginates to form the otic vesicle. The vesicle undergoes extensive morphogenesis, ultimately giving rise to the adult inner ear, an organ of exquisite complexity comprising distinct sensory, non-sensory and neuronal cell types of the auditory and vestibular apparatus. In humans, congenital hearing defects are often due to mutations in developmental genes. Thus, a mechanistic understanding of ear development not only provides insight into the molecular control of ear formation, but could also provide information relevant to the aetiology of human sensory disorders.

**Fig. 1.**
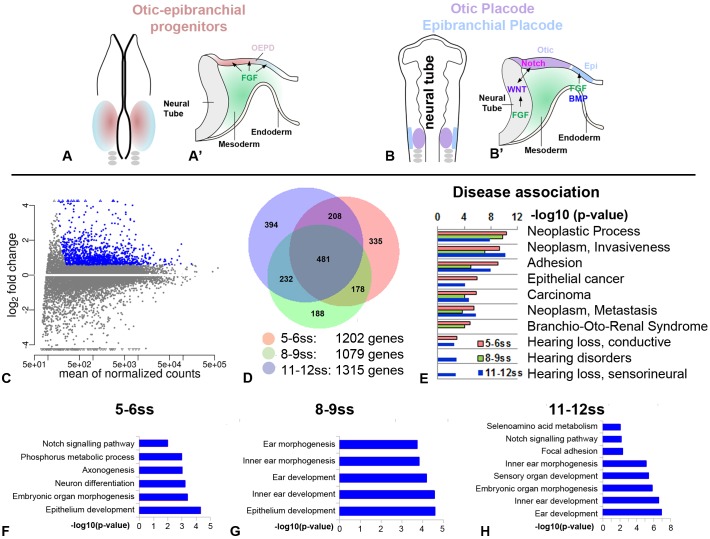
**Otic-enriched transcripts.** (A,B) Diagrams showing the location of OEPs at 5ss (A,A′, graded pink-blue) and the otic and epibranchial placodes at 11-12ss (B,B′; otic: purple; epibranchial: blue). OEPs are induced by mesoderm-derived FGFs (green in A′). Later, FGFs activate Wnt ligands in the neural tube, which cooperate with Notch to promote otic identity (B′), while FGFs and BMPs from the endoderm promote epibranchial fate. (C,D) RNAseq was performed on dissected otic placodes from 5-6ss, 8-9ss and 11-12ss and otic-enriched transcripts enriched were identified by comparison to the whole embryo (3ss); see also Tables S1 and S2. (C) Genes enriched in the otic placode at 5-6ss (blue; fold-change >1.5). (D) Venn diagram showing the number of otic-enriched genes at 5-6ss, 8-9ss and 11-12ss and their intersection. (E) Disease-association of otic-enriched genes. (F-H) Biological processes and signalling pathways over-represented in otic-enriched genes at each stage showing at most the five top over-represented terms for which *P*<0.01 (Fisher’s Exact test) for each category. Epi, epibranchial domain; OEPD, otic-epibranchial progenitor domain.

Specification towards otic fate occurs early in development and requires diverse signals and transcriptional inputs that act sequentially and/or in parallel. This process is initiated when sensory precursors in the pre-placodal region (PPR) become specified as otic-epibranchial progenitors (OEPs) under the influence of fibroblast growth factor (FGF) signalling (Fig. 1A; Ladher et al., 2000; Maroon et al., 2002; Martin and Groves, 2006; Nechiporuk et al., 2007; Nikaido et al., 2007; Phillips et al., 2001; Sun et al., 2007; Urness et al., 2010; Wright and Mansour, 2003). The OEP state is characterized by transcription factors like *Foxi1/3* (Khatri et al., 2014; Ohyama and Groves, 2004; Solomon et al., 2003), Dlx genes (Brown et al., 2005; Solomon and Fritz, 2002), *Pax2/8* (Christophorou et al., 2010; Freter et al., 2012; Hans et al., 2004; Mackereth et al., 2005) and *Spalt4* (Barembaum and Bronner-Fraser, 2007, 2010; Schlosser, 2006). After this step, Wnt and Notch pathways cooperate to promote otic and repress epibranchial character (Fig. 1B; Freter et al., 2008; Jayasena et al., 2008; Park and Saint-Jeannet, 2008; Shida et al., 2015). However, the transcriptional networks that control each step and the distinct differentiation programmes for otic and epibranchial cells are very poorly understood.

Here, we examine the active transcriptome of the developing ear from sensory progenitor to the overtly recognizable placode stage. Major changes occur as cells transit from a progenitor state to become OEPs, highlighting this as the most crucial step during otic induction. Time course analysis reveals previously unknown steps of otic commitment, defined by unique sets of transcription factors, and functional analysis not only reveals new downstream targets of known otic transcription factors, but also allows us to construct the first otic gene regulatory network (GRN) and predict connections therein. Its hierarchical organization reveals how, starting from a few factors initiated by otic induction, information is propagated through the network using positive feedback and feed-forward loops to stabilize otic identity and generate diversity by segregating otic and epibranchial fates.

## RESULTS

### New genes in otic placode development

**Fig. 2.**
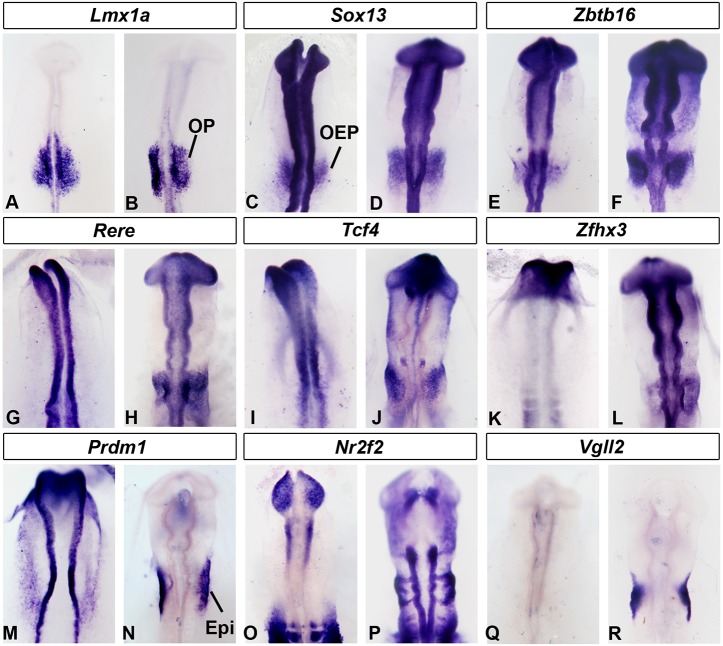
**Expression of transcription factors in the otic placode.** (A-R) *Lmx1a* (A,B), *Sox13* (C,D) and *Zbtb16* (E,F) are expressed in OEPs and in the otic placode (OP). *Rere* (G,H), *Tcf4* (I,J) and *Zfhx3* (K,L) expression starts at placode stages, whereas *Prdm1* is expressed in OEPs (M) but later restricted to the epibranchial territory (Epi) (N). At 12-13ss, *Nr2f2* (O,P) and *Vgll2* (Q,R) are absent from the otic placode, but present in epibranchial cells and the ventral ectoderm (*Vgll2*).

The progressive commitment of ectodermal cells towards otic identity occurs gradually, via a series of regulatory interactions that are not well understood. In avian embryos, otic specification begins around the 5-somite stage (ss) and by the 10ss, the otic ectoderm is already committed to its fate and to form an otic vesicle (Adam et al., 1998; Groves and Bronner-Fraser, 2000). To examine the steps leading up to this cell fate decision, we chose three time points for genome-wide transcriptome analysis, corresponding to the stages when (1) cells become specified as OEPs (5-6ss; Fig. 1A), (2) the placode acquires its characteristic thickened morphology (8-9ss) and (3) cells become committed to an otic fate (11-12ss; Fig. 1B).

To identify otic-enriched genes, we compared the otic transcriptome with that of whole embryos (3ss). Several hundred genes are enriched more than 1.5-fold at each stage (5-6ss: 1202 transcripts; 8-9ss: 1079 transcripts; 11-12ss: 1315 transcripts; Fig. 1C,D; Table S2). This analysis recovers many known otic transcription factors (23/27; e.g. *Pax2*, *Gata3*, *Gbx2*, *Foxg1*, *Eya1* and *Soho1*; Fig. S1A; Fig. S2). Functional annotation of otic-enriched transcripts (Fig. 1F-H) reveals progressive commitment to otic identity: ‘epithelium development’ is the most represented gene ontology (GO) term in OEPs (5-6ss; Fig. 1F), but this rapidly changes at 8-9ss and 11-12ss, when ‘inner ear development’ becomes the prominent term (Fig. 1G,H). Interrogating disease association databases reveals that transcripts enriched at the commitment stage are associated with hearing loss (Fig. 1E).

In total, this analysis identified 135 potential transcriptional regulators, of which 112 are novel with respect to the otic placode. To verify that they do represent otic-enriched transcripts, we assessed their expression using complementary methods. Twenty-three factors are indeed expressed in the otic placode according to the gene expression database GEISHA (http://geisha.arizona.edu/geisha/; Fig. S2). We found ten additional factors enriched in placode tissue as assessed by qPCR from dissected otic-epibranchial domains (Fig. S1B,C). Likewise, of 52 transcripts tested by NanoString 46 are present in the otic placode at 11-12ss with mean count >300 (Fig. S1D). For further validation, we performed *in situ* hybridization of 39 additional factors (Fig. 2; Figs S2, S3). This confirmed that the majority (34/39) is present, although not necessarily restricted to the otic placode. In summary, the transcriptome analysis identifies many genes not previously associated with ear development.

### Dynamic changes of gene expression in the developing otic placode

To capture changes in gene expression as otic cells mature, we used complementary approaches: *in situ* hybridization, transcriptome changes over time and hierarchical clustering. This allowed us to define synexpression groups and uncover distinct transcriptional states during otic placode maturation.

#### Analysis of transcription factor expression by *in situ* hybridization

*Lmx1a*, *Sox13* and *Zbtb16* are expressed in OEPs at 5-6ss and their expression persists in the otic placode until at least 12ss (Fig. 2A-F; Fig. S2). In contrast, *Zfhx3*, *Rere* and *Tcf4* (*Tcf7l2*) only become prominent in the otic territory from 10ss onwards (Fig. 2G-L; other factors: *Arid3*, *Atn1*, *Bach2*, *Klf8*, *Prep2*, *Tead3*, *Znf384*; Fig. S3I,J,O-AD; Fig. S2), whereas *Nr2f2*, *Vgll2* and *Klf7* are confined to the epibranchial region (Fig. 2O-R; Fig. S3K,L). *Prdm1* and *Tfap2e* transcripts change rapidly: they are broadly expressed at 5ss but then become restricted to the epibranchial territory after 9ss with *Tfap2e* also present in neural crest cells (Fig. 2M,N; Fig. S3M,N). In addition, several transcriptional regulators surround the otic placode at 11-13ss (Fig. S3AE-AJ), whereas others are widely expressed in the ectoderm including the otic territory (Fig. S3A-H; Fig. S2) or also present in neural crest cells (Fig. S3AJ-AN). We summarize the temporal and spatial expression of 95 known and new transcripts in Fig. S2.

#### Dramatic transcriptome changes accompany OEP specification

**Fig. 3.**
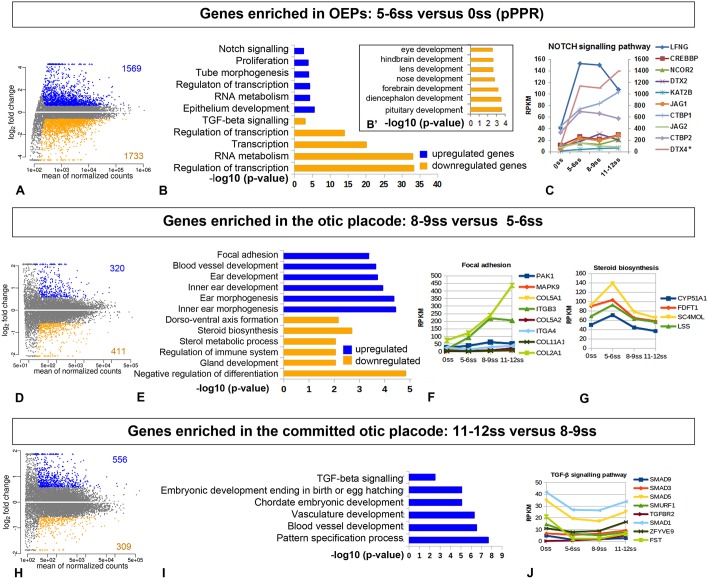
**Temporal changes in otic gene expression.** Pairwise comparison of the otic transcriptome at consecutive developmental stages: 5-6ss compared with 0ss (PPR; A-C), 8-9ss compared with 5-6ss (D-G) and 11-12ss compared with 8-9ss (H-J); see also Table S3. (A,D,H) Differentially expressed genes with a fold change >1.5; blue indicates upregulated transcripts; orange indicates downregulated transcripts. (B,E,I) Gene ontology analysis of up- and downregulated genes showing the five top over-represented biological processes or signalling pathway (*P*<0.01; Fisher’s Exact test). There is no significant association for the downregulated genes shown in H. (B′) At 5-6ss terms related to anterior structures are significantly under-represented relative to 0ss. (C,F,G,J) Changes of transcripts associated with signalling pathways over the entire time course. Asterisk indicates that the gene expression level is indicated by the *y*-axis on the right.

To highlight the main changes that occur at key steps of otic development, we performed pairwise comparisons of the otic transcriptome at consecutive stages: we compared (1) the PPR at 0ss with OEPs at 5-6ss; (2) OEPs at 5-6ss with the otic placode at 8-9ss; and finally (3) otic placodes at 8-9ss and at 11-12ss (Fig. 3A,D,H; Table S3). The most dramatic change occurs as cells transit from a sensory progenitor state in the PPR to specified OEPs, with 1569 transcripts being upregulated and 1733 downregulated at 5-6ss (Fig. 3A). Thereafter, changes occur more gradually (Fig. 3D,H). Transcripts associated with GO terms related to the acquisition of anterior character such as ‘eye, pituitary gland, nose, forebrain and diencephalon’ (*Pax6*, *Otx1/2*, *Mafa*, *Hesx1*, *Pax3*, *Dlx5*; Table S3) are significantly under-represented at the OEP stage (Fig. 3B,B′), consistent with the earlier suggestion that repression of anterior fate is an important step for otic induction (Bailey et al., 2006; Lleras-Forero and Streit, 2012).

#### Hierarchical clustering identifies distinct TF synexpression groups during otic commitment

**Fig. 4.**
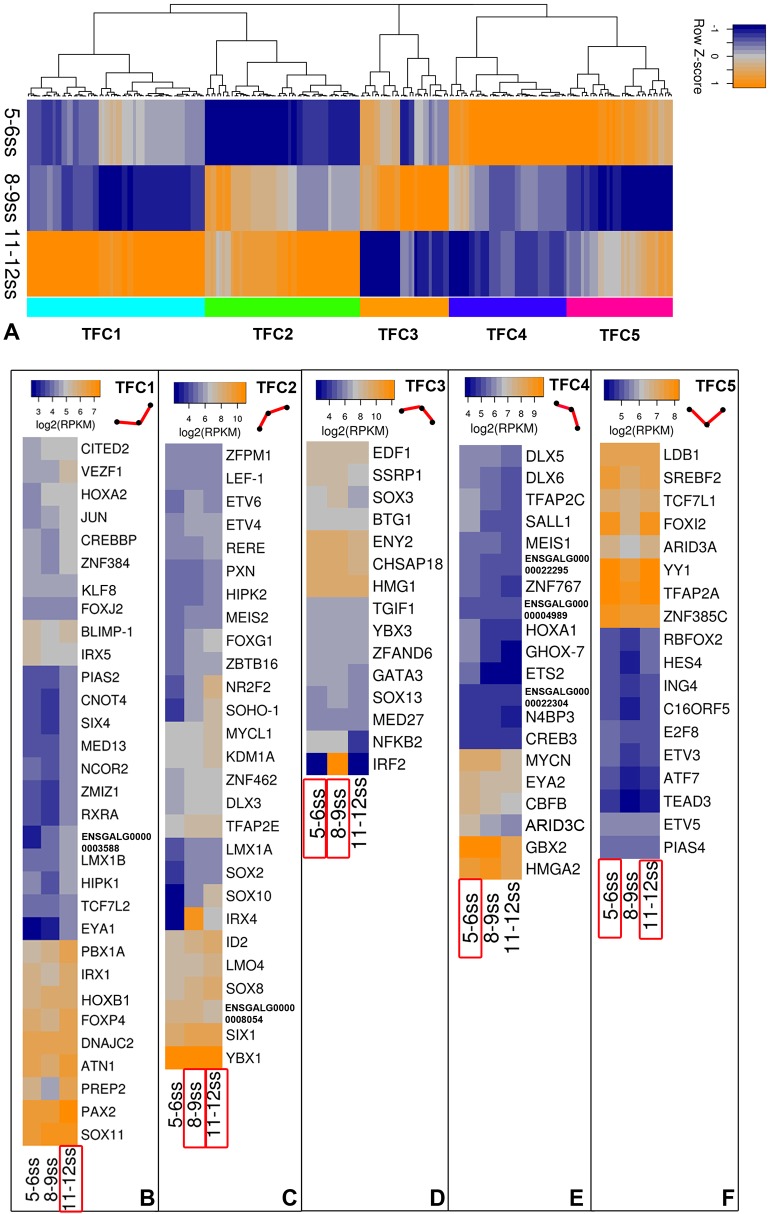
**Clusters of otic transcription factors.** (A) Otic transcription factors from the enrichment and time course analysis cluster into five clusters (TFC1-5) based on the row z-score of fold change relative to the PPR at 0ss. (B-F) Expression level of the top 50% transcription factors in each cluster. Line in the top right of each cluster represents the overall expression profiles across the three time points. See also Table S4.

Hierarchical clustering of all transcription factors that are either enriched in the otic placode compared with the whole embryo (Fig. 1) or differentially expressed over time (Fig. 3) reveals five major clusters denominated transcription factor cluster 1-5 (TFC1-5; Fig. 4A; Table S4). These clusters show distinct temporal profiles (Fig. 4A-F) and generally confirm our *in situ* hybridization data. For example, transcripts in TFC1 and TFC2 increase over time and these clusters include *Lmx1a*, *Zbtb16*, *Rere* and *Tcf4* (Fig. 2; Fig. S1B,C).

Combining these three approaches allows us to define distinct regulatory states as OEPs become committed to an otic fate. A number of PPR genes are reduced during OEP induction (*Dlx5/6*, *Irx1*, *Foxi3*, *Gbx2*; Fig. 4B; Fig. S2; see also Khatri et al., 2014), whereas a small group of transcripts [*Irx5*, *Lmx1a*, *cMyb* (Betancur et al., 2011), *Prdm1*, *Sall4* (Barembaum and Bronner-Fraser, 2007), *Sox13*, *Zbtb16* and *Znf385c*] becomes upregulated together with *Pax2* and *Etv4* (Figs 2, 4; Figs S2, S3). *Sox10* expression is initiated around 10ss together with *Foxg1* and *Dlx3* (Betancur et al., 2011; Khudyakov and Bronner-Fraser, 2009; Yang et al., 2013), and *Prdm1* becomes restricted to the epibranchial territory (Fig. 2), where it is co-expressed with *Pax2*, *Foxi2* and *Sox3* (Abu-Elmagd et al., 2001; Freter et al., 2008; Groves and Bronner-Fraser, 2000). Thus, already at the 10ss stage, otic and epibranchial progenitors begin to segregate and become molecularly distinct. As cells become committed to otic identity many new transcription factors start to be expressed (Figs 2, 4; Figs S2, S3) and the otic and epibranchial fates continue to diverge. In summary, our time course analysis reveals distinct regulatory states as cells acquire otic identity. Substantial transcriptome rearrangements occur within a brief period of only 6-8 h (from 1ss to 5ss) during the first step of otic induction: anterior character is inhibited and cells are specified as OEPs. Our analysis defines new factors that characterize a transcriptional state characteristic for OEPs at 4-5ss. As development proceeds, known ear-specific transcripts become more prominent, as do genes associated with hearing impairment, suggesting that our data might harbour new candidate deafness genes.

### Pathway analysis suggests potential novel regulators of otic placode formation

Signals from the surrounding tissues induce and pattern the otic placode. Although the role of FGF, Notch and Wnt pathways is well established (Abello et al., 2010; Freter et al., 2008; Ladher et al., 2000; Ohyama et al., 2006; Park and Saint-Jeannet, 2008; Phillips et al., 2004; Urness et al., 2010), it is likely that other signals are also involved. To explore this possibility, we used hierarchical clustering of all otic-enriched genes (from Fig. 1) together with differentially expressed genes from stage-wise comparisons (from Fig. 3) to generate six major clusters (denominated C1-C6; Fig. S4). Following pathway enrichment analysis for each cluster (Fig. S4A), we extracted the components of each significantly enriched or depleted pathway (Fig. 3C,F,G,J; Fig. S4B-D).

First, we evaluated pathways known to mediate otic development. As expected, Notch signalling components are present throughout placode formation and are over-represented in OEPs and in the placode (Fig. 3C; Fig. S4A,D) with *Lfng* expression increasing sharply as the placode forms and *Deltex2* and -*4* rising gradually. Wnt signalling components are highly enriched in cluster C2 (Fig. S4A,C) with the Wnt receptors *Fzd1-3* and mediators *Lef1* and *Tcf7l2* increasing steadily. In contrast, the Wnt antagonist *Sfrp2* drops sharply at 5-6ss. Components of the non-canonical Wnt pathway, such as *Wnt5a*, rise gradually together with *Rac1* and *Jun*, suggesting a role in placode assembly and morphogenesis. These findings are consistent with known changes in signalling events during otic commitment and therefore confirm the usefulness of this approach to predict the potential new pathways regulating otic development.

Next, we investigated whether new pathways emerge from this analysis. As OEPs become specified, components of the steroid biosynthesis pathway are markedly upregulated (Fig. 3E,G), whereas TGFβ signalling components drop sharply (Fig. 3I,J). Spliceosome components (Fig. S4A,B, cluster C2) peak at 5-6ss and 8-9ss. Consistent with this, spliceosomal defects are known to cause craniofacial disorders, some of which are associated with hearing loss (Lehalle et al., 2015). As a morphological placode forms, focal adhesion-related components become increasingly enriched, suggesting a role in placode assembly (Fig. 3E,F). These observations point to signals and pathways not previously associated with ear formation to explore in the future.

### Regulatory relationships reveal distinct transcriptional modules during otic commitment

Our time course analysis of gene expression predicts a transcriptional hierarchy during otic induction. To begin to test this hierarchy, we selected three transcription factors, Etv4, Pax2 and Lmx1a, for perturbation experiments for the following reasons. The transition from sensory progenitors to OEPs is mediated by the FGF pathway (Ladher et al., 2000; Maroon et al., 2002; Martin and Groves, 2006; Nechiporuk et al., 2007; Nikaido et al., 2007; Phillips et al., 2001; Sun et al., 2007; Urness et al., 2010; Wright and Mansour, 2003). Accordingly, the FGF mediator *Etv4* is expressed in OEPs (Lunn et al., 2007) and upregulated 7.5-fold from 1ss to 5-6ss (Table S3). Only ten transcription factors are strongly initiated at OEP stages (6- to 235-fold), with *Pax2* being the top factor (235-fold; Table S3), but *Lmx1a* is one of the few genes (6.3-fold; Table S3) exclusively expressed in otic, but not epibranchial cells (Fig. 2A,B). To explore the gene network downstream of these factors, we knocked down their expression by electroporation of antisense morpholino oligonucleotides at 1-2ss (MOs; Barembaum and Bronner-Fraser, 2010; Betancur et al., 2011; Christophorou et al., 2010). Experiments were assessed by NanoString nCounter at 10-12ss using a total of 216 probes including 70 otic genes (mostly transcription factors), markers for placode progenitors, other placodes, the neural plate, neural crest cells and non-neural ectoderm. This analysis provides a large-scale view of transcriptional changes in a single experiment enriching previously published data, which generally assessed a few genes at a time. In addition, selected transcripts were also assessed by RT-qPCR and/or *in situ* hybridization (Fig. 7; Fig. S6; Table S5). A gene was considered to be activated or repressed when its expression was reduced or enhanced after knockdown, respectively [NanoString: normalized mean count >300, ±1.2-fold change, adjusted *P*-value (*P*-adj)<0.1; RT-qPCR: ±1.5-fold change, *P*<0.05; *in situ* hybridization: absence or reduction of signal in electroporated cells]. These data allow us to add functional links between Etv4, Pax2, Lmx1a and other transcription factors in the otic GRN (Fig. 6), although cis-regulatory analysis will be required to distinguish between direct and indirect interactions. Although our experiments do not determine precisely when these interactions take place, we can infer this from our expression data, which show the onset of target genes.

#### Etv4 and Pax2 control the onset of OEP factors

**Fig. 5.**
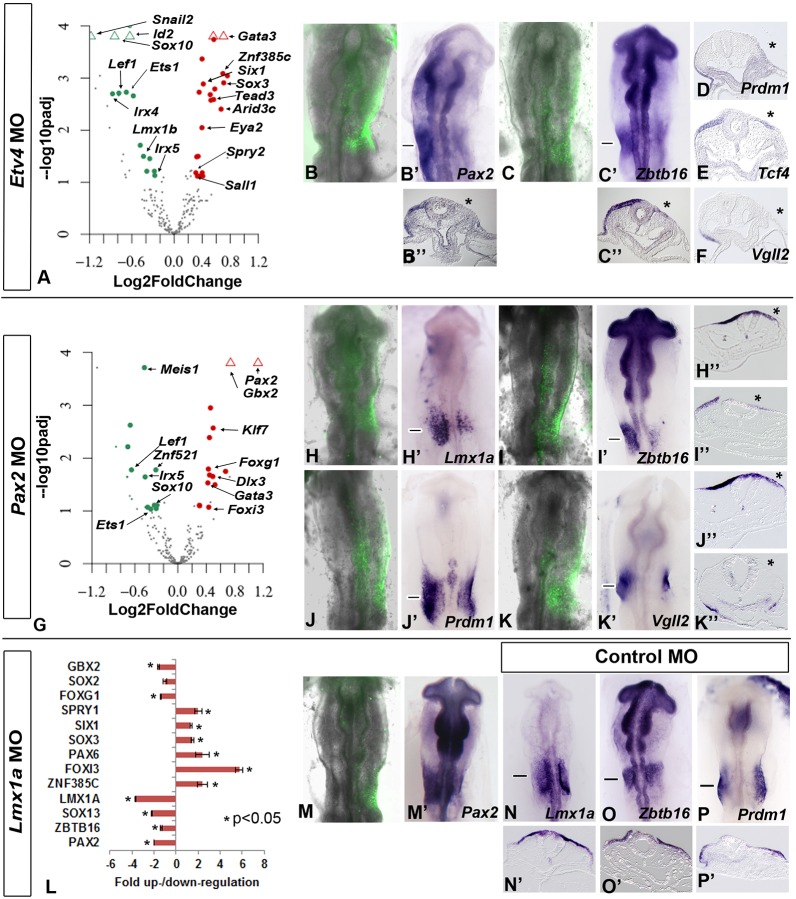
**Regulation of otic transcription factors by Pax2, Etv4 and Lmx1a.** Target-specific morpholinos were electroporated at 0-1ss and changes in gene expression were assessed by NanoString with three biological replicates, each of which containing five pieces of otic placode (A,G; see also Table S5), *in situ* hybridization (B-B″,C-C″,D-F,H′-K″,M,M′) or RT-PCR with two biological replicates each containing five pieces of otic placode (L). (A) Etv4 knockdown analysed by NanoString. Green indicates downregulated genes, red indicates upregulated genes. Open triangles represent data points that have a value beyond the axis limit. (B-F) *In situ* hybridization after Etv4 knockdown for the genes indicated in each panel. A reduction of *Pax2* (8/12; B′), *Zbtb16* (6/6; C′); *Prdm1* (7/11; D), *Tcf4* (8/10; E) and *Vgll2* (4/5; F) is observed. Asterisks indicate the electroporated side. B and C show morpholino fluorescence of the embryos shown in B′ and C′, respectively; B″ and C″ show sections through the embryos shown in B′ and C′, respectively, at the level marked by the horizontal lines. (D-F) Sections of embryos electroporated with Etv4 morpholino. (G-K″) Pax2 knockdown analysed by NanoString (G). Green indicates downregulated genes, red indicates upregulated genes. Open triangles represent data points that have a value beyond the axis limit. (H-K″) *In situ* hybridization after Pax2 knockdown for the genes indicated in each panel. Asterisks indicate the electroporated side. *Lmx1a* (4/4; H′), *Zbtb16* (4/4; I′), *Prdm1* (3/4; J′) and *Vgll2* (4/4; K′) are reduced. H-K show morpholino fluorescence of the embryos shown in H′-K′; H″-K″ show sections through the embryos shown in H′-K′, respectively, at the level marked by the horizontal lines. (L) Lmx1a knockdown analysed by RT-qPCR. The results are presented as fold change ±s.d. and two-tailed Student's *t*-test was used to calculate *P*-value. (M) Morpholino fluorescence of the embryo shown in M′ (3/4). (N-P″). *In situ* hybridization after control morpholino electroporation for the genes indicated in the panels; N′-P′ show sections of the embryos in N-P, respectively, at the level marked by the horizontal lines. *In* *situ* hybridization for each gene was performed on four embryos electroporated with control morpholino.

Etv4 knockdown leads to a reduction of *Pax2* expression (Fig. 5B-B″; Fig. S6A,B) confirming a requirement of FGF activity for *Pax2* expression. In addition, Etv4 activates other early OEP transcripts (*Irx5*, *Prdm1*, *Zbtb16*, *Sall4*, *Sox8*; Fig. 5A-F; Barembaum and Bronner-Fraser, 2007; Yang et al., 2013), and is also required for genes present at placode stages (*Lef1*, *Lmx1b*, *Sox10*, *Tcf4*; Table S5). In contrast, Etv4 represses some PPR genes (*Six1*, *Eya2*), the OEP factor *Znf385c* and late otic placode transcripts (*Tead3*, *Arid3*, *Sall1*; Fig. S6A,B; Table S5).

Many Etv4 targets are also regulated by Pax2 (OEP transcripts: *Irx5*, *Prdm1*, *Zbtb16*, *Sall4*; placode genes: *Lef1*, *Lmx1b*, *Sox10*, *Tcf4*; Fig. 5G,I-J″; Fig. S6A,C; Table S5). In addition, Pax2 activates the Etv4-independent OEP genes *Lmx1a* (Fig. 5H-H″) and *Sox13*, the placode transcripts *Eya1*, *Meis1*, *Zfhx3* and *Znf521* and maintains the PPR factor *Six1*, while repressing the posterior PPR genes *Foxi3* and *Gbx2*, which are normally cleared from the placode as it matures, the trigeminal marker *Pax3* (Wakamatsu, 2011), late onset otic genes (*Foxg1*, *Dlx3*) and *Kfl7*, which is later expressed in the epibranchial region (Fig. S6A,C). Pax2 is also required for the epibranchial-specific factor *Vgll2* (Fig. 5K-K″). Electroporation of control morpholinos does not affect otic gene expression (Fig. 5N-P″). Thus, many inner ear transcription factors depend on Etv4 and/or Pax2 activity placing these factors at the top of the otic hierarchy.

#### Lmx1a and Pax2: a positive feedback loop that maintains OEP factors and represses alternative fates?

**Fig. 6.**
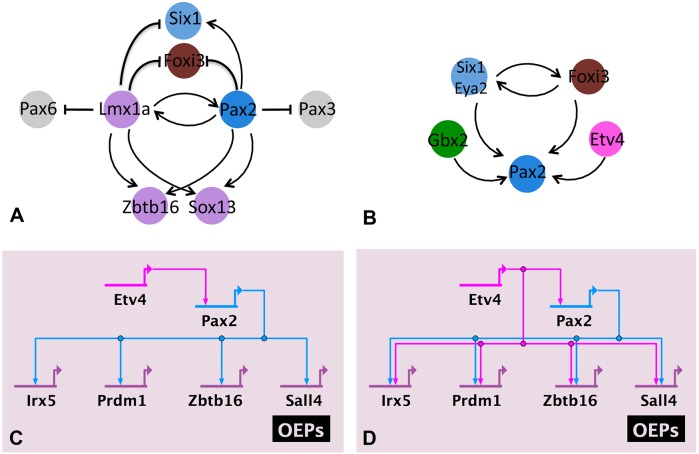
**Regulatory modules during OEP specification.** All diagrams summarize data from the literature and from this study (for details see text). (A) Lmx1a and Pax2 mutually activate each other, control common targets and appear to repress alternative fates (see text for details). (B) *Pax2* is controlled by the posterior PPR factors Six1, Eya2, Foxi3 and Gbx2, as well as by the FGF mediator Etv4. (C,D) Etv4 and Pax2 could act in a linear pathway (C) or in a feed-forward loop (D) to control other OEP genes (see text for details). *Irx5* is regulated by both Etv4 and Pax2; however, because *Pax2* is also regulated by Etv4 for simplicity the network in Fig. 7 assumes that Etv4 regulates *Irx5* via Pax2: Etv4→Pax2→Irx5.

Exploring Lmx1a function, we find that *Pax2* depends on Lmx1a input: *Pax2* expression is reduced when Lmx1a is knocked down (Fig. 5L-M′; Fig. S6A). Thus, they mutually regulate each other in a positive feedback loop and have common targets: both are required for *Sox13* and *Zbtb16* expression (Fig. 6A). In addition, Lmx1a is necessary for *Foxg1* and *Gbx2* expression. Like Pax2, Lmx1a also suppresses several transcripts (Fig. 5L; Fig. 6A), among them the PPR genes *Six1* and *Foxi3* and the lens/olfactory factor *Pax6*. Thus, together both transcription factors appear to promote OEP, but might also participate in the repression of alternative fates.

## DISCUSSION

Commitment to otic fate is initiated by the specification of OEPs from the posterior part of the pre-placodal region, followed by the acquisition of columnar placode morphology and, finally, placode invagination to form the otic vesicle. During this process, the otic territory is exposed to signals from surrounding tissues, and as morphogenetic events shape the embryo, its extrinsic environment changes constantly. As a result, ectodermal cells first initiate a transcriptional programme unique to otic progenitors and then form a patterned otic vesicle. Here, we have greatly expanded this transcriptional repertoire by identifying more than 100 factors that might be crucial for placode development, and are also new candidate genes for hearing impairment. Exploring the dynamic changes in gene expression over time together with perturbation analysis of selected transcription factors and integrating data from the literature (Fig. S5) allows us to propose the first GRN for otic commitment.

### A transcriptional mechanism for OEP specification

**Fig. 7.**
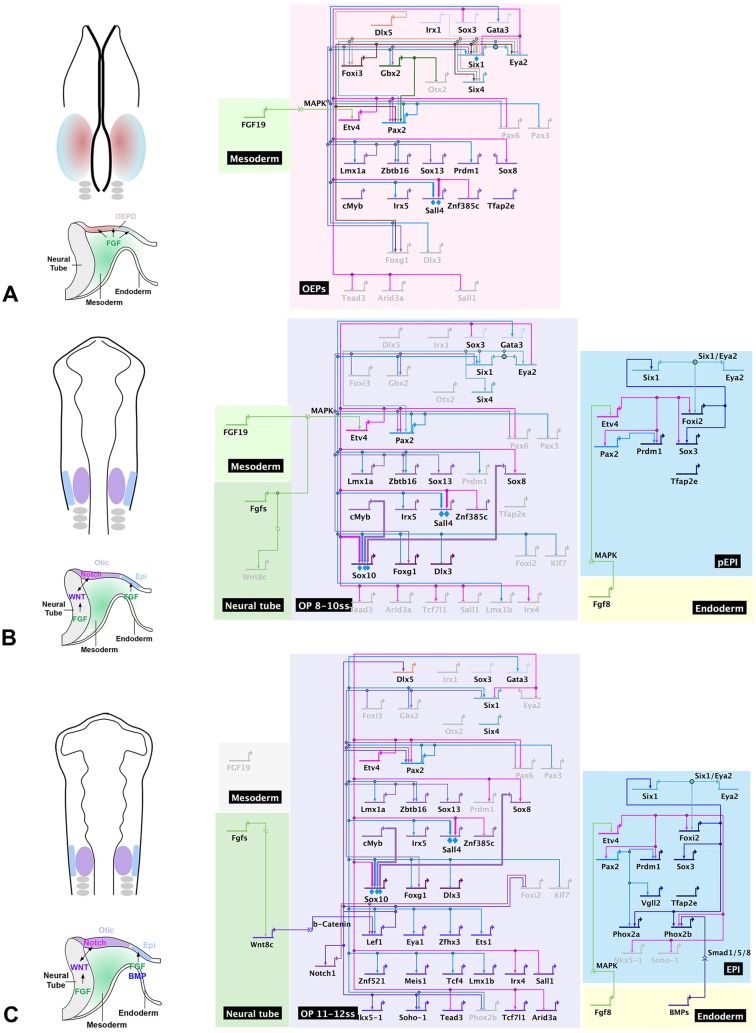
**Gene regulatory network incorporating new functional data.** (A-C) Signalling inputs, gene expression changes and regulatory relationships at the three different stages. Regulator links are based on data from the literature (Fig. S5) and our perturbation experiments (Fig. 5; Fig. S6A-C). Direct interactions confirmed from literature are indicated with blue diamonds and bold lines. As enhancers for most genes are currently unknown, the network assumes the simplest interactions depending on perturbation data (see also Fig. 6). Epi, epibranchial domain; OEPD, otic-epibranchial progenitor domain; OEPs, otic epibranchial progenitors; OP, otic placode; pEpi, pre-epibranchial domain.

To establish the first GRN that describes how sensory progenitor cells are committed to the inner ear lineage, we used a strategy that measures changes of all otic transcription factors after experimental perturbation using partial knockdown of three transcription factors (Fig. 7). This GRN has the deep structure characteristic of embryonic networks (Davidson, 2010), revealing the hierarchical organization of the process that drives otic commitment. Within this network, distinct transcriptional modules can be identified that gradually establish otic identity.

### The posterior PPR module

For the otic placode to develop, ectodermal cells must first go through a PPR state (Martin and Groves, 2006), which is identified by Six and Eya family members and by *Irx1* and *Dlx5/6*. Six1 is an important PPR determinant (Ahrens and Schlosser, 2005; Brugmann et al., 2004; Christophorou et al., 2009) and Irx1, Dlx5 and Foxi3 are known to regulate its expression (Glavic et al., 2004; Khatri et al., 2014; Pieper et al., 2012; Sato et al., 2010; Woda et al., 2003). In the posterior PPR, *Six1*, *Eya2*, *Gbx2* and *Foxi3* are crucial for otic placode formation (Brugmann et al., 2004; Christophorou et al., 2009; Solomon et al., 2003; Steventon et al., 2012). Gbx2 restricts the expression of *Otx2* to the anterior PPR (Steventon et al., 2012), whereas Foxi1/3 and the Six1/Eya2 complex regulate each other in a positive feedback loop (Khatri et al., 2014), perhaps to maintain posterior PPR identity. Together, all four proteins provide crucial input for the OEP transcription factor *Pax2* (Christophorou et al., 2010; Hans et al., 2004, 2007; Solomon et al., 2003, 2012): loss of Foxi1 function in fish and repression of Gbx2 and Six1 targets genes in *Xenopus* and chick, respectively, lead to the absence of *Pax2* (Fig. 6B; Fig. 7A). However, none of these transcription factors is sufficient to induce Pax2 in non-otic cells, suggesting that additional input is required.

### The OEP module

It is well established that FGF signalling induces OEPs from the posterior PPR (Ladher et al., 2000, 2005; Maroon et al., 2002; Martin and Groves, 2006; Nechiporuk et al., 2007; Nikaido et al., 2007; Phillips et al., 2001; Sun et al., 2007; Urness et al., 2010) and we show that the FGF target Etv4 is crucial for this process. *Etv4* is upregulated as posterior PPR cells transit to OEPs and is required for *Pax2* expression. Thus, a typical ‘AND’ gate controls *Pax2* in OEPs: its expression requires dual input from the posterior PPR factors Six1, Foxi3 and Gbx2 and from the FGF mediator Etv4 (Fig. 6B; Fig. 7A).

Our data suggest that Etv4 and Pax2 work in concert to promote otic identity and place them at the top of the transcriptional hierarchy for otic commitment (Fig. 7A). Together, they rapidly activate an OEP module consisting of *Sox8*, *Lmx1a*, *Zbtb16*, *Sox13*, *Prdm1*, *Sall4*, *Znf385c* and *Irx5*. The OEP module also contains the Pax2- and Etv4-independent factor *cMyb* (Betancur et al., 2011); however, its upstream regulators are currently unknown. As Etv4 and Pax2 share many targets, two scenarios could explain their mode of action. Etv4 might only regulate *Pax2*, which in turn controls other targets in a linear hierarchy (Fig. 6C; for simplicity, we depict this possibility in Fig. 7). However, it is equally possible that Etv4 and Pax2 act in a feed-forward loop, in which Etv4 is required for *Pax2* and both together control the expression of downstream targets (Fig. 6D) as is indeed the case for *Sall4* (Barembaum and Bronner-Fraser, 2007).

Although these and previous data implicate Pax2 as a key factor for otic specification and proliferation in chick (Christophorou et al., 2010; Freter et al., 2012), the mouse otic placode still forms in the absence of Pax2 (Burton et al., 2004; Favor et al., 1996; Torres et al., 1996) or Pax2 and Pax8 function (Bouchard et al., 2010). The fact that sauropsids have lost the *Pax8* gene (Christophorou et al., 2010; Freter et al., 2012) could explain the more prominent role of Pax2 in the otic GRN.

The newly identified OEP factors might play an important role in ‘locking’ cells in an OEP transcriptional state, where they are competent to respond to signals committing them towards otic and epibranchial fate. It has previously been suggested that continued FGF activity inhibits otic placode formation, while promoting epibranchial cells (Freter et al., 2008). Indeed, when chick OEPs are cultured in isolation they maintain otic identity from 5-6ss onwards and even generate neurons in the absence of additional signalling (Adamska et al., 2001; Groves and Bronner-Fraser, 2000). These findings suggest that in an OEP state ear precursors are FGF independent and we propose that the OEP module could be instrumental to maintain cell identity. Like Pax2 and Lmx1a, other OEP transcription factors might act in positive feedback loops to maintain OEP gene expression and repress alternative fates.

### Two distinct steps segregate otic and epibranchial progenitors

Our temporal analysis reveals two steps during the segregation of otic and epibranchial fates (Fig. 7A,B). Under continued FGF signalling, OEPs differentiate into epibranchial cells (Freter et al., 2008; Sun et al., 2007). This is consistent with our finding that Etv4 is required for epibranchial transcripts, as is Pax2. Dependent on the cellular context, Pax2 is likely to use different partners to impart cell fate (Kamachi and Kondoh, 2013; Stolt and Wegner, 2010): SoxE group members are prominent in otic cells, but they are absent from epibranchial placodes, where SoxB group members could represent major Pax2 partners (Ishii et al., 2001; Wood and Episkopou, 1999). In the otic lineage, expression of a small set of transcription factors (*Sox10*, *Foxg1* and *Dlx3*; Fig. 7B) is initiated downstream of Pax2 and the OEP module; for example, the Sox10 enhancer is directly controlled by Pax2, Sox8 and cMyb (Betancur et al., 2011), whereas epibranchial cells begin to initiate a different transcriptional programme.

In a second step, a large number of otic transcripts begin to be expressed, among them several that depend on canonical Wnt signalling from the neural tube (Freter et al., 2008; Ohyama et al., 2006) (Fig. 7C). Interestingly, FGF signalling, either directly or indirectly, promotes the Wnt mediators *Lef1* and *Tcf4*, suggesting that these upstream events might prime OEP cells for Wnt signalling. In contrast, under the influence of bone morphogenetic protein (BMP) signalling (Begbie et al., 1999) epibranchial progenitors continue to diverge from otic cells and the two fates become firmly established. In summary, the temporal resolution of our analysis highlights the complexity of cell fate decisions and reveals new transcriptional states as sensory progenitor acquire ear and epibranchial identity.

### The OEP module: a molecular circuit re-deployed in other organs?

Our analysis has defined a new transcriptional circuit, operating downstream of Pax2 and FGF/Etv4, which we propose is important for the specification of otic-epibranchial precursors. In addition to the ear, members of the OEP module are also co-expressed in the developing kidney and limb, including *Zbtb16* (Cook et al., 1995), *Lmx1* (Fernandez-Teran et al., 1997), *Prdm1* (Ha and Riddle, 2003) as well as *Sox8* (Chimal-Monroy et al., 2003) and *Sox13* (Kido et al., 1998; Wang et al., 2006)*.* Members of the Six and Eya families lie upstream of the OEP module, and together with Pax proteins are part of the fly retinal determination network (Salzer and Kumar, 2009), which in vertebrates also regulates the formation of other organs including the ear and kidney. Interestingly, the homologues of Zbtb16, Lmx1a and Prdm1 participate in fly eye formation although their relationship to the retinal determination network is unknown (Ko et al., 2006; Maeng et al., 2012; Wang et al., 2006). It is therefore tempting to speculate that the OEP module is part of an ancient sub-circuit that is re-deployed as a unit to govern cell fate decisions in different species and organs. The OEP module could provide an initial link between the top upstream regulators (Six/Eya and Pax proteins) that propagates information to the next level of the network.

### Uncovering new candidate deafness genes

Although much progress has been made recently to identify the genetic causes for hearing impairment in humans, many causative genes remain to be uncovered. In particular, mutations in developmental genes are associated with human deafness: for example Six1 or Eya1 mutations are known to cause Branchio-Oto-Renal Syndrome, an autosomal dominant disorder (Ruf et al., 2004). Functional annotation of our transcriptome data reveals a significant association with hearing loss, in particular for transcripts enriched in the committed otic placode (11-12ss; Fig. 1E). Indeed, the number of otic placode-enriched genes that fall into known human deafness loci is significantly higher than expected for a set of random genes (data not shown). These findings suggest that our data might harbour a number of novel candidates for congenital malformations of the ear and for hearing impairment.

In summary, integrating data from the literature (Fig. S5) and our new analysis has allowed us to establish the first gene regulatory network (Fig. 7) that models the early stages of ear development. By identifying key otic genes and the linkages between transcription factors at different levels of the network, we define new distinct regulatory states as cells acquire otic identity as well as the regulatory loops that stabilize cell fate decisions. In the future it will be important to identify the cis-regulatory elements controlling otic gene expression to determine direct and indirect interactions. The information gleaned from our analysis of otic transcriptional programmes will be crucial for future experiments aiming to reprogramme cells after loss of hearing and/or balance for the purpose of repair and regeneration.

## MATERIALS AND METHODS

### Tissue dissection and embryo experiments

Fertilized hens’ eggs (Winter Farm, Herts) were incubated in a humidified incubator at 38°C to reach the appropriate stage. For dissection, embryos were isolated from the egg and pinned out ventral side up on a resin plate in Tyrode's saline. The endoderm and mesoderm layers were removed using a G-31 syringe needle in the presence of a small volume of 10 mg/ml dispase before dissecting the future otic ectoderm based on the region expressing *Pax2* (Streit, 2002), rostral to the first somite and adjacent to future hindbrain rhombomeres 4-6.

For *in situ* hybridization, embryos were isolated in PBS, fixed in 4% paraformaldehyde in PBS and processed as described previously (Streit and Stern, 2001). Digoxigenin-labelled antisense RNA probes were synthesized with T7, T3 or SP6 RNA polymerase (Roche) as appropriate from expressed sequence tags, cloned fragments or previously published plasmids (Table S6).

For morpholino knockdown experiments, electroporation at 0-1ss was performed either unilaterally with control and experimental morpholino, or in the same embryo with control and experimental morpholino on different sides. *Pax2* and *Etv4* morpholinos were validated previously (Betancur et al., 2011; Christophorou et al., 2010); as controls, standard control morpholinos (5′-CCTCTTACCTCAGTTACAATTTATA-3′) were used. For *Lmx1a*, a splicing-blocking morpholino (5′-ACCCCCAGTGTCCCCATACCTTCCT-3′) targeting the exon 4-intron 4 junction was used and deletion of exon 4 was confirmed by RT-PCR followed by sequencing the PCR product. Changes in gene expression were assessed by whole-mount *in situ* hybridization or from five to ten dissected otic placodes by RT-qPCR or NanoString. For dissection, the non-electroporated or control morpholino electroporated side and the first somite is used as a guide.

### RNA isolation, library construction and sequencing

About 100 placodes were collected from each stage for RNAseq library preparation. Immediately after dissection, tissues were lysed in lysis buffer (Ambion) and RNA was isolated using the RNAqueous-Micro Kit (Ambion). Libraries were prepared with TrueSeq RNA Sample Preparation V2 kit (Illumina) and sequenced with Illumina HiSeq 2000 (Illumina) to 1×50 cycles or HiSeq 2500 to 2×100 cycles.

### Sequence alignment

Reads ware aligned with TopHat2 (v2.0.7) to Ensembl chick genome Galgal4.71 (Kim et al., 2013). Transcripts for individual samples were assembled with Cufflinks (v2.1.1) (Trapnell et al., 2010), with combined Ensembl gene annotation (Galgal4.71.gtf) and Refseq acquired from UCSC table browser as a guide. All assemblies were merged to obtain a merged annotation file, which was passed to Cuffdiff (v2.1.1) to obtain normalized RPKM value for each gene, and to easyRNAseq (v2.1.0) to retrieve the count table for all genes (Delhomme et al., 2012). All sequencing data were deposited in Gene Expression Omnibus (GEO) under accession number GSE69185.

### Identification and analysis of differentially expressed genes

DEseq (v1.16.0) was used to identify enriched otic genes relative to the whole embryo (Anders and Huber, 2010) based on the count table generated above. A gene was considered to be expressed in the otic region when the normalized RPKM was >4, and the number of normalized counts was >300. Owing to a lack of biological replicates, ‘Blind’ mode was used in DEseq, which treats the two samples to be compared as replicates to estimate the variance of gene expression. This approach assumes that most genes are not differentially expressed (as would be the case in biological replicates), thus generally overestimating variance, because the variance among samples from different conditions is usually larger than among biological replicates. Therefore, this method produces very conservative results with small numbers of differentially expressed genes (Anders and Huber, 2010). Using *P*-adj<0.1 and a fold change of >1.5, only a fraction of known otic genes (14/37) is recovered (Table S1). To maximize the discovery of otic placode-enriched genes, all genes with a fold change of >1.5 were included as candidates for further analysis. This analysis recovers 27/37 known otic genes. Validation by *in situ* hybridization was used as a secondary screen to ensure that the gene list did indeed represent otic placode-enriched transcripts. Indeed, of all 39 factors screened by *in situ* hybridization only five are not expressed in the placode, validating this as a useful strategy to identify genes enriched in otic cells.

Gene ontology analysis for differentially expressed genes was performed with DAVID (DAVID Bioinformatics Resources 6.7, http://david.abcc.ncifcrf.gov/) (Huang da et al., 2009a,b). Disease-related enrichment was analysed with Webgestalt (Wang et al., 2013; Zhang et al., 2005).

### Partitioning of otic genes into different clusters

Both enriched otic genes and differentially expressed transcription factors from pairwise analysis (except downregulated genes at 5-6ss relative to 0ss) were used for hierarchical cluster analysis. Fold change of these factors at 5-6ss, 8-9ss and 11-12ss relative to 0ss was transformed into row z-score with heatmap.2 and corresponding heatmaps were generated using gplot within R (R Core Team, 2012; Warnes, 2012). Otic genes were clustered in the same way to generate the clusters in Fig. S4.

### Quantitative RT-PCR and NanoString nCounter analysis

RNA from dissected otic tissue and the whole embryo was isolated using the RNAqueous-Micro Kit (Ambion) and reverse transcribed. Primers for target genes were designed with PrimerQuest (IDT). qPCR was performed in technical triplicates using Rotor-Gene Q (Qiagen) with SYBR green master mix (Roche). The ΔΔCt method was used to calculate the fold change, which was expressed as FC=2^∼(−ΔΔCt)^ (Livak and Schmittgen, 2001). *Gapdh*, *Hprt* and *Rplp1* were used as reference genes to calculate the fold change. RT-qPCR to validate the otic-enriched genes was performed from a single biological replicate, and RT-qPCR for morpholino experiments used two biological replicates; the *P*-value generated from a two-tailed Student's *t*-test was used to evaluate the statistical significance.

NanoString analysis was performed in triplicate per experimental condition with the nCounter Analysis System using a customized probe set of 216 genes. Five to ten tissues were lysed in lysis buffer (Ambion) and total RNA was hybridized with capture and reporter probes according to the nCounter Gene Expression Assay manual. The results were analysed using the raw count with DEseq2 (Love et al., 2014). A transcript was considered dysregulated when the mean count was >300, up- or downregulated by at least |log2foldchange|>log2(1.2) and the *P*-adj<0.1. The cut-off of mean count 300 is empirical based on the observation that genes not expressed or very weakly expressed in the otic region had a count of less than 300.

### GRN construction

GRN construction was performed manually using BioTapestry. Different regions of the network were defined according to the known biology of OEP induction, segregation of otic and epibranchial domains and the signalling input from adjacent tissues taking into account the new transcriptional states identified in this study and data from the literature (Fig. S5). Genes were allocated to each region based on their temporal and spatial expression pattern (published or determined in this study). Interactions were plotted according to published data from different species as summarized in Fig. 5; these largely depend on the analysis of mutants or knockdown experiments using a few genes as otic markers and, with few exceptions, lack enhancer information. The model presented in Fig. 7 incorporates the data from the current study, which determined changes in gene expression after MO knockdown of three transcription factors by RT-qPCR, NanoString nCounter and/or whole-mount *in situ* hybridization. For some transcripts all three methods were used, whereas for others only one or two approaches were employed (Fig. S6). Occasionally, we observed a discrepancy between the three detection methods; in this case, an interaction was defined if expression change was detected by *in situ* hybridization, or if two methods provided the same result. NanoString nCounter evaluation allows the analysis of hundreds of genes in the same sample, thus providing a global view of gene expression changes.

To establish links between upstream regulators and their downstream targets, unless otherwise stated we assumed the most parsimonious pathway: e.g. if gene 1 regulates gene 2 and 3, and gene 2 regulates gene 3 we assumed the simplest explanation of gene 1 gene 2 gene 3.
